# Minor hepatectomy for hepatocellular carcinoma in a patient with portal hypertension: A case report and review of the literature

**DOI:** 10.1097/MD.0000000000032176

**Published:** 2022-12-02

**Authors:** Ting-Chun Tseng, Wen-Yao Yin

**Affiliations:** a School of Medicine, Tzu Chi University, Hualien, Taiwan; b Department of Surgery, Dalin Tzu Chi General Hospital, Buddhist Tzu Chi Medical Foundation, Chiayi, Taiwan.

**Keywords:** hepatocellular carcinoma, liver resection, liver transplantation, portal hypertension

## Abstract

Curative modalities for early hepatocellular carcinoma (HCC) include liver resection (LR) and transplantation. For patients with portal hypertension (PH), liver transplantation (LT) is the preferred treatment but is oftentimes limited by organ shortage and can lead candidates to drop off due to disease progression, while hepatectomy has a higher risk of complications. This would pose a dilemma as to whether wait for donor organs or prioritize hepatectomy.

**Patient concerns:**

The patient was a 56-year-old male, a case of liver cirrhosis due to hepatitis C with sustained virological response following direct-acting antiviral agents. He was a liver transplant candidate, presented to the gastroenterology outpatient department for a recently-diagnosed liver tumor during a regular follow-up session. Pre-operative survey revealed PH manifested by thrombocytopenia, splenomegaly, huge splenorenal shunt and varices. The patient’s Child-Pugh score was 7.

**Interventions and diagnosis:**

Considering the patient’s overall condition, tumor size and location, and a shortage of grafts, he underwent segment 5 and 6 partial hepatectomy. The pathological diagnosis was moderately differentiated HCC.

**Outcomes:**

His postoperative course was complicated by refractory intraabdominal infection (IAI) and recovered under aggressive antibiotics treatment. He remained recurrence-free for over a year.

**Conclusion:**

For patients with early resectable HCC, the approach of having a minor hepatectomy followed by salvage transplantation should serve as a compromising strategy. Tumor resection retards the progression of the disease. Comprehensive healthcare can expectantly improve clinical outcomes.

## 1. Introduction

Hepatocellular carcinoma (HCC) is an aggressive, primary liver tumor that usually arises in the context of chronic liver disease and cirrhosis. It is one of the most common malignancy and the third leading cause of death worldwide.^[1]^

The curative-intent therapies for small, solitary HCC (tumor size of less than 5 cm) remain at issue, especially in patients with portal hypertension (PH).^[2]^ Radiofrequency ablation (RFA) is limited by tumor size (less than 3 cm), location, and lower rates of complete ablation.^[2,3]^ Liver transplantation (LT) is regarded as the best treatment, but there are a limited number of liver donors. Liver resection (LR) has seen an impressive technical growth in recent years, but is still burdened by relatively high rates of post-operative complications.^[4]^ Although there is an increased risk following the surgery, the long-term outcome for HCC patients with PH remains uncertain;^[5–7]^ whether surgical resection should be restricted to such patients is still a matter of controversy.^[8,9]^

Herein, we report a case in which segment 5 and 6 partial hepatectomy was performed in a patient diagnosed as early stage HCC with pre-operative clinically significant PH manifested by thrombocytopenia, splenomegaly, splenorenal shunt and varices.

## 2. Case report

A 56-year-old Chinese man, a liver transplant candidate, presented to the gastroenterology outpatient department for a recently-diagnosed liver tumor during a regular follow-up session. The patient had a history of hepatitis C virus-associated liver cirrhosis was treated with direct-acting antiviral agents one year beforehand and achieved sustained virological response, type 2 diabetes mellitus under insulin control, and cholecystectomy for gallbladder stone complicated with bile leakage and intraabdominal infection (IAI). The patient denied any family history of malignant tumors.

**Figure 1.**
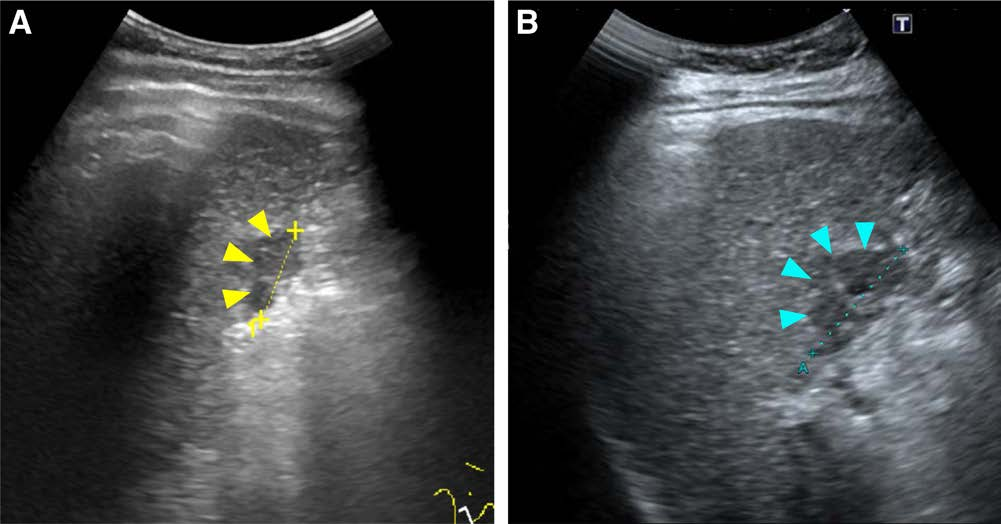
Ultrasound follow-up of the hepatic tumor. Abdominal ultrasonography showed an enlarging hypoechoic mass near segment 5 of the liver, measuring 24 mm (A, yellow arrowhead) and 35 mm (B, blue arrowhead) within three months.

On gastroenterology outpatient follow-up, he presented with no symptoms and signs and denied any discomforts. He had no jaundice or icteric sclera. No ascites and encephalopathy were detected. General examination was unremarkable. Ultrasound imaging detected an enlarged, hypoechoic nodule at segment 5, from largest diameter of 24 to 35 millimeters in three months (Fig. 1). Therefore, he was transferred to hepatology surgeon for evaluation and surgical intervention. Laboratory tests showed white blood cell of 4610/μL, platelet count of 85,000/μL, serum albumin of 3.2 g/dL, total bilirubin of 2.5 mg/dL, %prothrombin time of 117%, and indocyanine green retention test at 15 min of 75% (normal: less than 10% retention after 15 min of injection). The Child-Pugh score was 7, grade B. The albumin-bilirubin (ALBI) score/modified ALBI grade was –1.64/2a.

**Figure 2.**
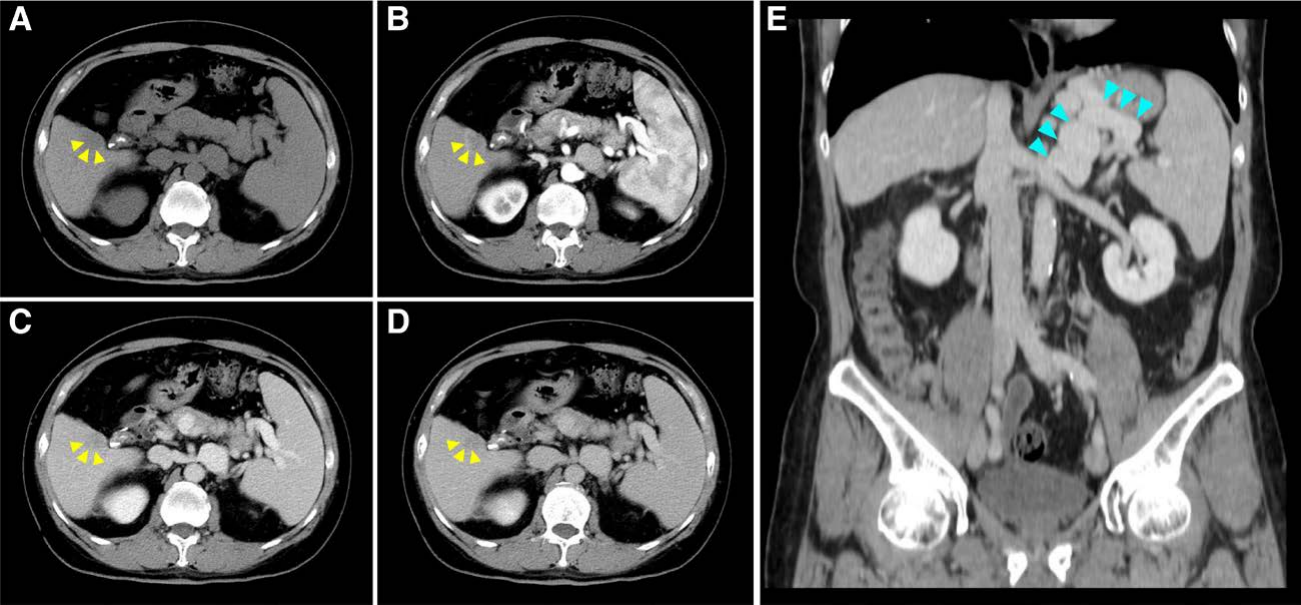
Triple-phase abdomen and pelvis computed tomography before partial hepatectomy. (A) Pre-contrast. (B) Arterial phase. (C) Portal phase. (D) Delayed phase of computed tomography showed an early enhance and early washout nodule (yellow arrowhead) in segment 5 of the liver, compatible with hepatocellular carcinoma. (E) Splenomegaly measured by size of 13.95 cm and a huge splenorenal shunt and varices (blue arrowhead) were clearly demonstrated in coronal plane.

Upper gastrointestinal endoscopy showed no obvious lesions. Triple-phase computed tomography (CT) scan of the abdomen demonstrated a heterogeneous, tumor of 3.5 cm in size in segment 5 near liver surface with early enhancement on the arterial phase with rapid washout of contrast on the portal phase. Clinical staging was T2N0M0, according to American Joint Committee on Cancer (AJCC) 2017. Barcelona Clinic Liver Cancer (BCLC) staging: stage A3. It also showed liver cirrhosis, splenomegaly up to 13.95 cm, huge splenorenal shunt and varices (Fig. 2). There was no evidence of the metastasis of tumor on whole body CT. Combined with the patient’s medical history, the final diagnosis was malignant neoplasm of liver 3.5 cm at segment 5, suspected HCC (T2N0M0, BCLC stage A3).

Intraoperative sonography showed the tumor was located chiefly in segment 5 and a small part in segment 6. An experienced gastrointestinal surgeon performed partial resection of both segment 5 and 6 of liver. The pathological diagnosis of the tumor was moderately differentiated HCC measuring 28 mm × 25 mm × 18 mm. The nearest resection margin was 6 mm. The pathological staging was T1bN0M0, according to American Joint Committee on Cancer (AJCC) 8th edition. Fibrosis grade was F4.

As planned, the patient was admitted to the intensive care unit following surgery. Flomoxef sodium was prescribed as empirical antibiotic. During the treatment period, the bacterial culture of drainage fluid performed on postoperative day (POD) 7, detecting carbapenem-resistant *Pseudomonas aeruginosa* (CRPA), *Escherichia coli* (*E. coli*), *Enterococcus faecalis* (*E. faecalis*). According to the culture and sensitivity report, Vancomycin, Ceftazidime, and Metronidazole were prescribed.

On POD 13, the patient had increasing amount of turbid, subhepatic drainage accompanied with fever up to 39.6 °C. Laboratory showed %Seg of 80.4%, CRP of 2.07 mg/dL, albumin of 2.4 mg/dL. Abdominal CT showed post-operative change at the S5 liver with parenchymal abscess. CT guided drainage was done and showed turbid fluid. Fourth week after operation, drainage fluid again grew CRPA, *E. coli*, *E. faecalis*. Antibiotics were thus switched to Gentamicin, Ceftazidime, and Metronidazole advised by infectious disease specialist. Thereafter, the patient had an uneventful recovery and was discharged on POD 65.

On 1-month and 3-month post-discharge outpatient follow-up, small amounts of persistently purulent drainage were still noted. Therefore, the patient was readmitted for aggressive antibiotic treatment for IAI and removal of drainage tube if remission is obtained. Bacterial culture of drainage fluid was obtained and again detected CRPA and *E. coli*. Finally, drains were removed under appropriate antibiotics coverage. Subsequent treatment plans were Bisoprolol for PH control, Furosemide and Lactulose for liver cirrhosis, and awaiting LT. Upon 12 months postoperatively, he has held on to free of infection and recurrence HCC for at least one-year follow-up.

## 3. Discussion

Early HCC clinical assessments are necessary to determine the appropriate treatment modality (i.e., RFA, LT, LR). Such assessments include liver function, tumor staging, surgical approach, and the presence of comorbidities and PH.^[10,11]^ Despite prevalent staging systems stratifies patients according to the characteristics of the tumor, underlying liver disease and performance status,^[12]^ none of them take the myriad of factors that may affect patients with HCC into account.^[13,14]^

Kutlu et al analyzed a total of 1894 patients with HCC measuring up to 50 mm treated with RFA, LT, or LR. For patients with lesions measuring >30 mm, RFA was significantly associated with worse disease-specific survival compared with LR and LT, suggesting the maximum HCC tumor size cutoff to be 30 mm.^[3]^ In our case, RFA was relative inapplicable due to tumor size exceeding 30 mm, tumor location near liver hilum, and possible severe hilar adhesion associated with previous bile leakage after cholecystectomy.

When comparing LR and LT, studies found that the overall survival and the recurrence-free survival rates for LT were generally better than LR for HCC patients with Child A^[15]^ and Child B cirrhosis.^[16,17]^ As studies showed that life expectancy was worse for salvage LT compared to primary LT,^[18,19]^ primary surgery should therefore be the ideal choice of treatment, even when the tumor is resectable. However, given the shortage of organs, LR is regarded as a reasonable first-line treatment for patients with small HCC and good liver function, alongside a secondary LT as an option in case of recurrence.^[20]^ These previous studies supported our patient, a liver transplant candidate, to benefit from primary LR with salvage LT as the second-line therapy.

Patients with clinically significant PH (i.e., characterized pathophysiologically by the presence of either esophagogastric varices, portosystemic shunt, thrombocytopenia due to splenomegaly, or an increased hepatic venous pressure gradient by 10 mm Hg) are susceptible to diverse complications and markedly reduced life expectancy.^[18]^ In our case, platelet counts of 85,000/μL, splenomegaly, splenorenal shunt and varices were significant clinical features for PH even without pressure gradient measurement. European Association for the Study of the Liver (EASL) guideline believe that clinically significant PH is not an absolute contraindication, suggesting patients with MELD score of less than 9 have a lower risk of liver decompensation. The BCLC classification recommends LR only in early tumor stages and patient without PH; emerging evidence is still in need for the decision-making for eligibility for LR.^[11,12,21]^

Limited research compared the outcomes of LT and LR for HCC patients with PH. One study included 38 patients with very early HCC (under 2 cm), well liver function, and PH, suggesting LR can provide a satisfactory recurrence-free survival.^[22]^ On the other hand, several studies suggested that preoperative clinically significant PH is related to an increased incidence of severe postoperative morbidity (odds ratio [OR]: 1.66), 90-day surgical mortality (OR: 1.77‐3.70), and 5-year mortality (OR 1.29‐2.07) following LR; and consequently, LT is the optimal procedure for patients with PH and liver decompensation within the Milan criteria.^[5,6]^ However, transplant waitlist grew dismally by the day, causing an approximate 20% of candidates to drop out eventually due to tumor progression and/or deteriorated liver function. The Asia demographic subgroup has seen a greater dropout rate.^[23,24]^ Accordingly, emerging studies suggested that selected patients with esophageal varices^[25]^ or platelet count no less than 105,000/μL^[26]^ may also benefit from LR with fair morbidity and mortality rates.

LR is burdened by relatively high rates of postoperative morbidity (approximately 5%‐40%),^[4]^ which could exert a great impact on the long-term clinical course. Posthepatectomy infective complications occurred in up to 15% of patients,^[27]^ and among them, IAI, with an incidence of 9%, is a commonly encountered and severe type of complication.^[28]^ As infectious and high-grade complications were significantly associated with long-term outcome and cancer-specific mortality,^[26]^ controllable factors were crucial in preventing such adverse events. Intraoperatively, major hepatectomy (more than three segments) or prolonged operation time was identified as independent factors for major complications,^[26,29,30]^ and postoperative nutritional support and broad-spectrum anti-infectious treatment may serve as an effective strategy for improving long-term survival.^[31,32]^ In our case, there was repeated purulent drainage and severe infection, but with attentive care, the patient surmounted the postoperative clinical course.

In brief, as seen in selected clinical scenarios (i.e., patients with borderline liver conditions, minimal comorbidity, or no other treatment options available), minor hepatectomy with adequate healthcare should be considered despite the risk of adverse outcomes. Physicians should discuss and inform the patient of the advantages and disadvantages of LT and LR. Further research can investigate and clarify the subgroups of patients with PH who could benefit the most from surgical resection.

## 4. Conclusion

We have reported a case of early-stage HCC and clinically significant PH that underwent hepatectomy with refractory postoperative infectious complications. We believe that despite patients with PH increased clinical risks, with adequate healthcare, minor hepatectomy should still be considered performed in regions that lack availability or unequal access to deceased donor organs.

## Acknowledgements

The authors are grateful to Ching-Yu Tseng for editing the manuscript.

## Author contributions

**Conceptualization:** Ting-Chun Tseng, Wen Yao Yin.

**Data curation:** Ting-Chun Tseng, Wen Yao Yin.

**Supervision:** Wen Yao Yin.

**Writing – original draft:** Ting-Chun Tseng.

**Writing – review &amp; editing:** Ting-Chun Tseng, Wen Yao Yin.
